# Impact of general vs. neuraxial anesthesia on neonatal outcomes in non-elective cesarean sections

**DOI:** 10.3389/fped.2025.1518456

**Published:** 2025-03-03

**Authors:** Enrico Cocchi, Rita Pini, Antonella Gallipoli, Marcello Stella, Patrizio Antonazzo, Federico Marchetti, Vanni Agnoletti

**Affiliations:** ^1^ AUSL Romagna, Neonatal and Pediatric Intensive Care Unit, Bufalini Hospital, Cesena, Italy; ^2^ Department of Medical and Surgical Sciences (DIMEC), Alma Mater Studiorum—University of Bologna, Bologna, Italy; ^3^ Department of Precision Medicine and Genomics, Columbia University, New York, NY, United States; ^4^ AUSL Romagna, Anesthesiology and Intensive Care Unit, Bufalini Hospital, Cesena, Italy; ^5^ AUSL Romagna, Obstetrics and Ginecology, Bufalini Hospital, Cesena, Italy; ^6^ AUSL Romagna, Pediatric and Neonatal Intensive Care Unit, Santa Maria Delle Croci Hospital, Ravenna, Italy

**Keywords:** anesthesia, general anesthesia, cesarean section, neonatal outcome, neonatal resuscitation, inverse probability of treatment weighting (IPTW), respiratory

## Abstract

**Background:**

Cesarean section is a common surgical procedure, usually performed under neuraxial anesthesia and, more rarely, under general anesthesia. The choice of anesthesia in cesarean sections can significantly influence neonatal outcomes, especially in urgent and emergency cases. Previous studies have shown mixed results, often confounded by the inclusion of both elective and emergency cesarean section cases, varying statistical methods, and a focus solely on resuscitation immediate-term neonatal outcomes.

**Objective:**

This study aims to use robust statistical methods to evaluate the impact of anesthesia type on immediate and longer-term neonatal outcomes in urgent and emergency cesarean section cases, where additional detrimental factors might influence this relationship.

**Methods:**

We analyzed 395 women who underwent non-elective cesarean sections between 2021 and 2023. Inverse probability of treatment weighting (IPTW) served to focus on the role of anesthesia type eliminating confounding variables effect, in simulated randomized controlled trial conditions.

**Results:**

General anesthesia increases odds of neonatal resuscitation (OR 6.1, *p* < 0.001), NICU admission (OR 1.8, *p*: 0.04), and a 15% lower Apgar score at 1 min (*p*: 0.02). General anesthesia also increased NICU admission rate for respiratory insufficiency (OR 7.6, *p* < 0.001), the need for oxygen (OR 4.8, *p*: 0.003) and CPAP (OR 3.6, *p* < 0.001) in NICU. Negative controls and consistent sensitivity analyses further validated the robustness of our findings.

**Conclusion:**

General anesthesia in non-elective cesarean sections is associated with worse neonatal outcomes, extending beyond the resuscitation phase to sustained NICU morbidity. Our study provides novel insights into the specific neonatal resuscitation maneuvers required when general anesthesia is used, enhancing clinicians preparedness for managing high-risk deliveries. These findings underscore the critical importance of anesthesia choice, advocate for the preference of neuraxial techniques, and highlight the need for further research into long-term neonatal outcomes.

## 1 Introduction

Cesarean section (CS) is a common surgical procedure, typically performed under neuraxial anesthesia (NA), which encompasses both spinal and epidural techniques, and less frequently under general anesthesia (GA) (1, 2). The choice of anesthesia can significantly influence both maternal and neonatal outcomes (3, 4). While NA is generally preferred due to its lower risk profile and better outcomes for both mother and child, GA is sometimes necessary or preferred by the anesthesiologist, particularly in urgent or emergency situations (5, 6).

The impact of anesthesia type on neonatal outcomes in these high-stakes scenarios remains a critical area of investigation. Previous studies have explored the effects of different anesthesia techniques on neonatal outcomes with mixed results. Some studies have indicated that GA is associated with poorer immediate neonatal outcomes, such as lower Apgar scores and increased need for resuscitation (3, 7). However, these studies often include a mix of elective and non-elective (urgent or emergency) CS cases, potentially confounding the results.

In this view, the urgency level, driven by acute fetal or placental conditions, may itself contribute to the detrimental effects of GA on the newborn, making it difficult to isolate the true impact of the anesthesia type (8, 9). Furthermore, the use of GA in elective CS is exceedingly rare due to its well-documented adverse maternal outcomes. As a result, the disproportionately low utilization rate leads to highly imbalanced cohorts, which can substantially increase the risk of bias in analysis results, even when applying rigorous statistical methods. Additionally, while immediate neonatal outcomes are important, there is a growing recognition of the need to understand the longer-term implications of anesthesia choice, including NICU admission rates and subsequent diagnoses and treatments required (10).

Our study aims to address these gaps by focusing exclusively on urgent and emergency CS cases, providing a clearer picture of the impact of GA compared to NA in high-risk, time-sensitive situations where GA is more frequently used (5). By employing a robust analytical approach, including inverse probability of treatment weighting (IPTW), we aim to specifically isolate the effect of anesthesia type on neonatal outcomes.

IPTW is increasingly used in all branches of medicine to overcome the limitations of observational retrospective studies by reproducing the conditions of randomized controlled trials (11, 12). This method allow to specifically focus on a single treatment variable - anesthesia type in our study - by eliminating the potential confounding effects of all other variables and cohort selection. To the best of our knowledge, IPTW has only been used once in this context for elective-only CS, providing further evidence of GA detrimental role on immediate neonatal outcomes (13).

We aimed to conduct a more nuanced analysis of the effects of GA on newborns in a properly balanced cohort of urgent/emergency CS, where additional detrimental factors might influence the relationship between anesthesia type and neonatal outcomes. Furthermore, we examined neonatal outcomes beyond the immediate post-delivery period, providing a deeper and comprehensive insight into which specific neonatal outcomes and characteristics are adversely affected by GA.

## 2 Methods

### 2.1 Study design and setting

This is a retrospective cohort collected at Maurizio Bufalini Hospital (Cesena, Italy), a tertiary care center, from January 1, 2021, to December 31, 2023. We included women who underwent urgent or emergency CS during this period. Elective cases were excluded from the study to specifically focus on urgent and emergency scenarios, thereby minimizing potential biases arising from the substantial imbalance in the distribution of the treatment variable.

### 2.2 Study population

A total of 395 women who underwent non-elective (urgent or emergency) CS over the 3-year period were included in the study, accounting for 37.5% of all cesarean sections performed at our hospital in that time frame (*n* = 1053). Among these, 355 (89.9%) received NA and 40 (10.1%) received GA. Only complete cases were included in the analysis to minimize potential biases and ensure the robustness of the results. Characteristics of the cohort under study according to anesthesia type are reported in Table 1. During the 3-year study period, three neonates died before hospital discharge following cesarean section delivery. These newborns were all extremely premature, with a gestational age below 27 weeks. Due to their distinct characteristics in terms of gestational age, birth weight, and other clinical parameters, we excluded them from the cohort to prevent potential bias in the analysis. In accordance with clinical guidelines, emergency CS were defined as those requiring a decision-to-delivery time <30 min due to immediate life-threatening conditions, such as persistent fetal bradycardia, placental abruption, uterine rupture, umbilical cord prolapse, or twin delivery complications. Urgent CS were defined as those requiring a decision-to-delivery time <75 min, generally for maternal or fetal compromise that is not immediately life-threatening, such as dystocia, breech presentation in labor, or maternal complications precluding vaginal delivery.

**Table 1:** Characteristics of the cohort under analysis and their stratification with the treatment variable (anesthesia type) used in inverse probability of treatment weighted (IPTW) regression.

Continuous variables	General anesthesia			
	no (n. 355, 89.9%)	yes (n. 40, 10.1%)	Significance	
	Median [Q1—Q3]	Median [Q1—Q3]	*p*-value	IPTW *p*-value
BMI	23.8 [21.1–27.15]	23.3 [20.95–25.12]	0.17/	0.7
Age	33 [(30–37)]	33.5 [29–37.25]	0.6/	
GA	39 [(37–40)]	39 [36.25–40]	0.1/	0.9
Weight	3,325 [(2,840–3,690)]	2,997.5 [2,086.25–3,713.75]	0.06	0.6
Apgar 1	9 [(9–9)]	8.5 [(6–9)]	<0.001***	
Apgar 5	10 [(10–10)]	10 [(8–10)]	0.001**	
Categorical variables	*n* (%)	*n* (%)	*p*	IPTW *p*-value
IUGR	34 (9.58%)	8 (20%)	0.06^†^	0.8
Placental alteration	7 (1.97%)	2 (5%)	0.23/	
Previous CS	77 (21.69%)	6 (15%)	0.4/	
Emergency CS	20 (5.63%)	25 (62.5%)	<0.001***	0.9
Sex	179 (50.42%)	22 (55%)	0.6/	
Gestational hypertension	28 (7.89%)	2 (5%)	0.8/	
Gestational diabetes (ins)	18 (5.07%)	1 (2.5%)	0.7/	
Gestational diabetes	49 (13.8%)	5 (12.5%)	1/	
Gestational glucose intolerance	54 (15.21%)	5 (12.5%)	0.8/	
Labour analgesia	209 (58.87%)	13 (32.5%)	0.002**	0.9
CS TOLAC refusal	33 (9.3%)	3 (7.5%)	1/	
CS fetal reason	110 (30.99%)	16 (40%)	0.3/	
CS due to abruptio placentae	12 (3.38%)	13 (32.5%)	<0.001***	0.9
Decision-to-delivery time (minutes)	14 [(6–22)]	2 [(1–4)]	<0.001***	0.4
Parity			0.4/	
Nulliparous	249 (70.14%)	24 (60%)		
Primiparous	83 (23.38%)	13 (32.5%)		
Multiparous	23 (6.48%)	3 (7.5%)		
PMA	41 (11.55%)	6 (15%)	0.6/	
CS maternal reason	21 (5.92%)	0 (0%)	0.23/	

All listed variables were considered as possible covariates in the IPTW regression analysis. Variables that showed a univariate regression association *p*-value <0.2 (as reported in the “*p-value*” column) were included in the analysis to eliminate their potential confounding effect on the treatment variable (anesthesia type). The “*IPTW p-value*” column reports the association of the covariate with the treatment variable after IPTW weights were applied, demonstrating the robustness of the performed analysis in eliminating such effects in the pseudo-cohort on which regression models were run, as shown by the lack of association among these variables and the treatment variable in the “*IPTW p-value*” column. The same statistics are reported for each outcome variable under analysis in the Supplementary Table 1.

^/^ means *p* > 0.2.

^†^ means *p* ≤ 0.2.

* means *p* ≤ 0.05.

** means *p* ≤ 0.01.

*** means *p* ≤ 0.001.

### 2.3 Data collection

Data were collected in accordance with the hospital-approved study protocol, as approved by the hospital ethics committee, for which included patients provided specific informed consent. The variables collected included:

- Maternal age
- Maternal Body Mass Index (BMI)
- Parity (nulliparous vs. primiparous vs. multiparous)
- Previous CS history
- Gestational hypertension
- Gestational diabetes with insulin-dependence
- Gestational diabetes, non-insulin-dependence
- Gestational glucose intolerance
- Pregnancy onset (spontaneous vs. medically assisted)
- Presence of intrauterine growth restriction (IUGR)
- Presence of placental abnormalities (placenta previa, accreta, increta, percreta)
- Gestational age (GSA)
- Newborn weight
- Newborn sex
- CS urgency level (urgent vs. emergency)
- Decision-to-delivery time (minute)
- Epidural analgesia in labour before CS (analgesia conversion)

Specific indications for CS were also included. Full list of included variables and their stratification by anesthesia type are reported in Table 1. Supplementary Table 1 reports their stratification with each outcome variable.

### 2.4 Anesthesia type

NA encompasses both spinal and epidural techniques. The majority of NA cases utilized the spinal technique, whereas epidural anesthesia was administered via an epidural catheter already placed for labor analgesia upon request (analgesia conversion). Only cases where GA followed established guidelines, employing rapid sequence induction and intravenous administration of opioids (Fentanyl/Sufentanil), anesthetics (Propofol), and muscle relaxants (Succinylcholine/Rocuronium), were included in the analysis (14).

### 2.5 Outcomes

The primary neonatal outcomes assessed were:

1. Need for neonatal resuscitation immediately following delivery
2. Apgar score at 1 and 5 min
3. NICU admission

The secondary outcomes analysis included a more detailed examination of specific resuscitation maneuvers required immediately after delivery, such as positive pressure ventilation (PPV), endotracheal intubation (ET), chest compressions (CPR), and administration of adrenaline. We also focused on specific neonatal diagnoses and treatments during the NICU stay. To ensure that the identified differences were related to anesthesia type and not to factors that had a longer-term influence, we categorized the main diagnoses for NICU admission into neonatal asphyxia and respiratory issues. Asphyxia was defined according to national guidelines as low Apgar scores, prolonged resuscitation, metabolic acidosis (arterial cord pH <7.0 and/or base deficit ≥12 mmol/L), and evidence of encephalopathy not attributable to other causes. We used asphyxia as a negative control, as in cases of urgent or emergency CS, it is generally due to detrimental factors that began long before anesthesia administration. Additionally, we further divided respiratory diagnoses into distress vs. insufficiency. NICU treatments were analyzed in terms of respiratory support needs, including oxygen (O_2_), continuous positive airway pressure (CPAP), and mechanical ventilation (MV). We used central catheter insertion as another negative control, with the same rationale as for asphyxia: to validate our results, we did not expect to find any significant difference in this variable when stratified by anesthesia type.

### 2.6 Statistical analysis

To account for potential confounders and simulate the conditions of a randomized controlled trial, we used IPTW-weighted regression models (15). IPTW generates a pseudo-cohort in which all included variables are well balanced between treatment groups, effectively eliminating the influence of these covariates and enabling a focused investigation of the treatment variable effect on the outcome. In line with the IPTW approach, we included only covariates that were present before the treatment was administered and were related to both the treatment and/or the outcome under analysis. All variables listed in Table 1 were considered as potential initial covariates, and we performed a univariate association analysis with each outcome under study and with anesthesia type. The results of these associations are reported in Table 1; Supplementary Table 1. Due to non-normal distribution, tested using the Kolmogorov–Smirnov test, continuous variables are presented as median and interquartile range, while categorical variables are presented as category-wise percentages. Consistent with statistical literature on IPTW-weighted regression models variable selection (16–18), variables that showed a univariate *p* < 0.2 (as reported in Table 1) with the treatment - anesthesia type - or the outcome variable were included in the model. Logistic regression was used to evaluate binary outcomes such as the need for neonatal resuscitation and NICU admission. Due to their count nature and clear visual distribution, Poisson regression was used to analyze count outcomes such as Apgar scores at 1 and 5 min. To ensure the robustness of our findings, we also performed the following sensitivity analyses:

1. Random effects mixed models for year of delivery and anesthetist code to account for temporal and provider-related variations
2. Aggregated variables analysis, where all variables included in each model - once grouped as: primary vs. secondary outcomes (in terms of NICU diagnoses and treatment) were considered as an overall covariate pool for each outcome tested.

## 3 Results

### 3.1 Primary outcomes

**Figure 1:**
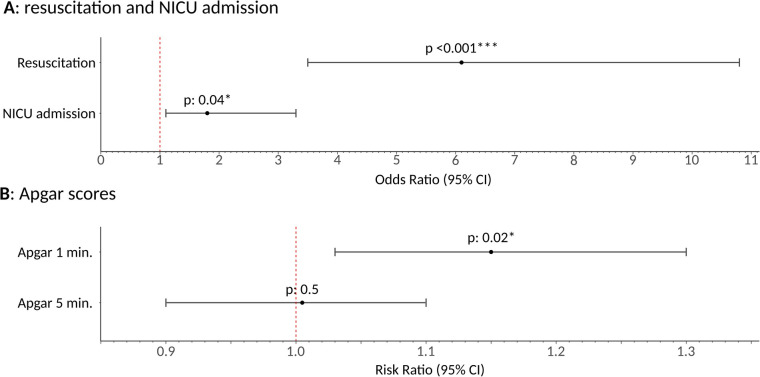
Graphical representation of inverse probability of treatment weighting regression model results of general anesthesia effect on the primary outcomes under analysis. Plot **(A)** shows odds ratio and relative 95% confidence interval, alongside association *p* values. Plot **(B)** reports risk ratio and relative 95% confidence interval, alongside association *p* values. General anesthesia, once isolated from the effect of all other potential confounding covariates, was found to increase the need for newborn resuscitation [OR 6.1 (3.5–10.8), *p* < 0.001] and NICU admission (OR 1.8 [1.1–3.3, *p*: 0.04). Additionally, it was shown to reduce the Apgar score at 1 min by 15% [RR 1.15 (1.1–1.3), *p*: 0.02], while no effect was detected on the apgar score at 5 min (*p*: 0.5), likely due to the effectiveness of neonatologists resuscitation efforts. * means *p* ≤ 0.05; ** means *p* ≤ 0.01; *** means *p* ≤ 0.001.

IPTW-weighted regression models revealed a significant impact of GA on several primary outcomes: the need for newborn resuscitation [OR 6.1 (3.5–10.8), *p* < 0.001], NICU admission [OR 1.8 (1.1–3.3), *p*: 0.04], and Apgar score at 1 min [RR 1.15 (1.03–1.3), *p* 0.02]. To increase clarity of results presentation, the latter is presented as an inverse RR, indicating that a value greater than 1 reflects a detrimental effect on the Apgar score. No significant effect was detected on the Apgar score at 5 min [RR 1.03 (0.9–1.1), *p*: 0.5]. Results are graphically summarized in Figure 1; Table 1 provides the stratification and both univariate association and IPTW balanced *p*-values for potential covariates and the treatment variables, while Supplementary Table 1 reports the same statistics with outcome variables. This illustrates which variables had a univariate association *p* < 0.2 and were subsequently included in the models, and demonstrates the effectiveness of IPTW weighting in nullifying significant associations between covariates and the treatment and outcome variables under analysis. For clarity, Apgar scores are divided with a threshold of 7 in the Supplementary Table 1, although univariate and regression analyses treated these scores as continuous variables. Table 2 presents the stratification and both univariate association and IPTW-weighted regression *p*-values for the outcome variables relative to the treatment variable. All statistical analyses were performed using R version 4.3.3, IPTW analysis was performed using “*riptw*” package (19).

**Table 2:** Characteristics and regression statistics between the treatment variable (anesthesia type) and the outcome variables used for the inverse probability of treatment weighted (IPTW) regression.

Outcome variable	General anesthesia			
	no (n. 355, 89.9%)	yes (n. 40, 10.1%)	Significance	
	*n* (%)	*n* (%)	*p*-value	Regression *p*-value
Primary outcomes				
Newborn resuscitation	60 (16.9%)	17 (42.5%)	<0.001***	<0.001***
Newborn NICU admission	64 (18.03%)	16 (40%)	0.003**	0.04*
Apgar 1 min	35 (9.86%)	11 (27.5%)	0.003**	0.02**
Apgar 5 min	7 (1.97%)	4 (10%)	0.02*	0.5
Secondary outcomes				
Newborn resuscitation				
Positive pressure ventilation	68 (19.15%)	17 (42.5%)	0.002**	<0.001***
Endotracheal intubation	7 (1.97%)	4 (10%)	0.02*	0.02*
Cardio-pulmonary Resuscitation	1 (0.28%)	0 (0%)	1/	1/
Adrenaline administration	1 (0.28%)	0 (0%)	1/	1/
NICU diagnosis				
Newborn asphyxia	6 (1.69%)	3 (7.5%)	0.05^†^	0.8
Newborn respiratory alteration	41 (11.55%)	9 (22.5%)	0.07^†^	0.008**
Newborn respiratory distress	29 (8.17%)	5 (12.5%)	0.4/	0.4
Newborn respiratory insufficiency	7 (1.97%)	3 (7.5%)	0.07^†^	<0.001***
NICU treatment				
Oxygen administration	11 (3.1%)	5 (12.5%)	0.02*	0.003**
CPAP administration	34 (9.58%)	9 (22.5%)	0.03*	<0.001***
Mechanical ventilation	13 (3.66%)	3 (7.5%)	0.2/	0.05
Central catheter placement	14 (3.94%)	4 (10%)	0.1	0.8

The “*p*-value” column reports univariate regression analysis *p*-value, while the “regression *p*-value” column reports the final IPTW model results (as reported in Figures) of the association between the treatment and each outcome variable, once the effect of the remaining potential confounding variables was eliminated through IPTW regression. This allows evaluation of covariates confounding effect on the association that IPTW eliminates, shaping the association strength between treatment and outcome analysis.

^/^ means *p* > 0.2.

^†^ means *p* ≤ 0.2.

* means *p* ≤ 0.05.

** means *p* ≤ 0.01.

*** means p ≤ 0.001.

### 3.2 Secondary outcomes

Variables showing a univariate association *p* < 0.2 with the treatment and/or secondary outcome variable are detailed in the Supplementary Materials, supplementary results section.

#### 3.2.1 Resuscitation steps

**Figure 2:**
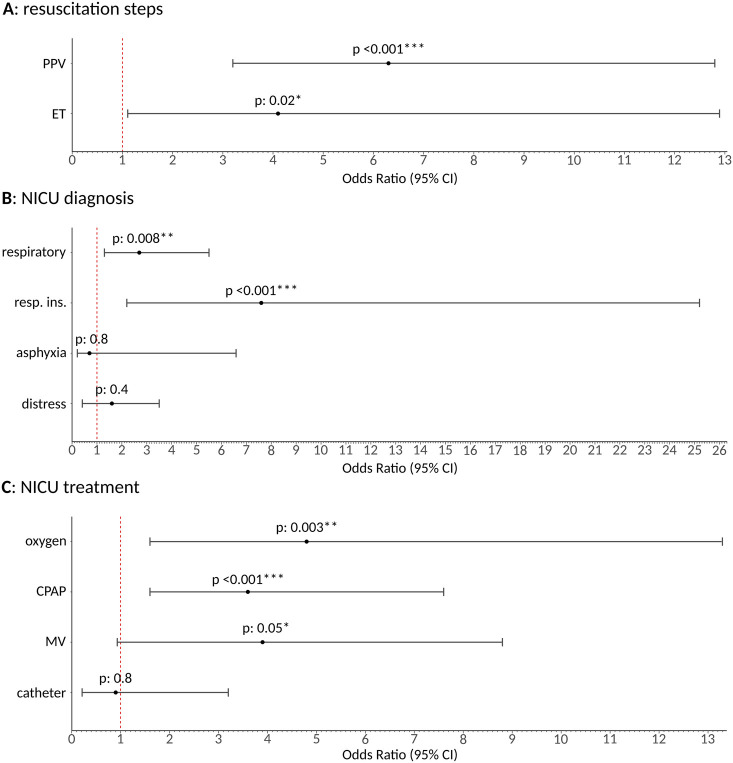
Graphical representation of inverse probability of treatment weighting regression model results of general anesthesia effect on the secondary outcomes under analysis as resulting odds ratio and relative 95% confidence interval. **(A)** Shows the impact on specific neonatal resuscitation steps (PPV, positive pressure ventilation; ET, endotracheal intubation). **(B)** Illustrates the effects on NICU admission diagnoses. **(C)** Depicts the effect on NICU treatments, including oxygen, continuous positive airway pressure (CPAP), mechanical ventilation (MV) administration, and central catheter placement (negative control). General anesthesia, once isolated from the effect of all other potential confounding covariates, significantly increased the need for PPV and ET in the immediate post-delivery phase. It also led to a higher NICU admission rate for respiratory diagnoses, particularly respiratory insufficiency, and increased the need for oxygen and CPAP in the NICU, with a trend observed for mechanical ventilation. No significant effect was identifiable on asphyxia and central catheter placement, as expected, since these were used as negative controls, further reinforcing the robustness of our findings. / means *p* > 0.2; * means *p* ≤ 0.05; ** means *p* ≤ 0.01; *** means *p* ≤ 0.001.

IPTW regression models indicated a significant effect of GA on PPV [OR 6.3 (3.2–12.8), *p* < 0.001] and ET [OR 4.1 (1.1–12.9), *p*: 0.02]. No significant association was found with CPR and adrenaline administration, likely due to the low number of newborns requiring these procedures in our cohort. Significant associations are graphically depicted in Figure 2A.

#### 3.2.2 NICU admission diagnosis

A significant effect of GA on respiratory diagnosis [OR 2.7 (1.3–5.5), *p* = 0.008] was identified through IPTW. This was particularly related to respiratory insufficiency [OR 7.6 (2.2–25.2), *p* < 0.001], consistent with the expected effects of GA on newborns. No significant effect was found for respiratory distress alone (*p*: 0.4) or asphyxia (*p*: 0.8), the latter serving as a negative control. Results are graphically presented in Figure 2B.

#### 3.2.3 NICU treatment

IPTW regression models showed a significant effect of GA on oxygen administration [OR 4.8 (1.6–13.3), *p*: 0.003] and CPAP [OR 3.6 (1.6–7.6), *p* < 0.001] in the NICU. A trend towards significance was observed for mechanical ventilation [OR 3.9 (0.9–8.8), *p*: 0.05], which likely did not reach significance due to the relatively low number of patients requiring mechanical ventilation in our cohort. No significant association was found with central catheter placement (*p*: 0.8), used as a negative control. Results are graphically summarized in Figure 2C.

### 3.3 Sensitivity analysis

The sensitivity analysis performed, both aggregated and random models, confirmed our findings. GA mixed random and aggregated models showed comparable levels of odds/risk ratio and statistical significance for both primary and secondary outcomes, except for NICU admission and respiratory diagnosis that showed a *p*: 0.01 in both random and combined models, oxygen supplementation that showed a *p*: 0.003 in the aggregated model, and mechanical ventilation, which reached statistical significance (*p*: 0.04), in the aggregated model. Thus, this was the only variable that showed a significance trend in the main and random models and reached statistical significance in the aggregated one. Overall, the comparability of results further attests to the robustness of our analysis.

## 4 Discussion

In this study, we aimed to evaluate the impact of GA on neonatal outcomes in non-elective CS. Our primary outcomes included the need for neonatal resuscitation, Apgar scores at 1 and 5 min, and NICU admission rates. Secondary outcomes focused on specific resuscitation maneuvers, detailed NICU treatments, and neonatal diagnoses. Our findings indicate that GA is associated with significantly worse neonatal outcomes compared to NA, not only in the immediate post-delivery phase but also in the longer term.

Our results align with previous studies that have shown GA to be associated with poorer neonatal outcomes (3, 7). However, our study extends these findings by specifically focusing on urgent and emergency CS cases, where the impact of anesthesia type might be more pronounced due to the critical nature of these deliveries. Additionally, the more frequent use of GA in such scenarios results in a more balanced cohort, allowing for a more robust comparative analysis. Through IPTW, which to the best of our knowledge has never been applied in this setting, we specifically focused on the role of a single treatment variable (anesthesia type) while eliminating the effects of other potential confounding factors, providing robust evidence of the detrimental impact of GA on neonatal outcomes in urgent or emergency CS.

The need for neonatal resuscitation, including specific maneuvers such as PPV and ET, was significantly higher in the GA group (Figures 1A, 2A). This finding is critical as it underscores the immediate adverse effects of GA on neonatal health and highlights the need for neonatologists to be well-prepared. Additionally, GA was associated with higher NICU admission rates, especially for respiratory insufficiency (Figure 2B). IPTW also revealed an increased need for specific NICU treatments in the GA group, such as oxygen, CPAP, and mechanical ventilation—where a trend was identified—further emphasizing the increased morbidity associated with GA (Figure 2C).

While it is well-established that GA suppresses neonatal respiratory function in the immediate post-delivery phase, increasing resuscitation needs and lowering Apgar scores, our findings reveal that its detrimental effects in non-elective CS extend beyond delivery room outcomes. These include increased NICU admissions and the need for more intensive interventions, highlighting a sustained impact on neonatal health that warrants further investigation into long-term outcomes. Additionally, our study provides novel insights by detailing specific resuscitation maneuvers required in this setting. This granularity enhances understanding of the immediate challenges posed by GA and equips clinicians with actionable knowledge to optimize neonatal care in high-risk deliveries.

We also studied the effect of GA on asphyxia and central catheter insertion, using them as negative controls, as we expected no role on those. No significant correlation was found with these outcomes, reinforcing the robustness of our findings (Figures 2B,C). Overall, our results strongly suggest that GA detrimentally affects both immediate and longer-term neonatal outcomes, potentially due to the additional effects of GA drugs in urgent settings. All sensitivity analyses, including random effect mixed models that account for temporal and operator-sensitive differences, and aggregated outcomes, confirmed the strength of the identified associations across different variables, thereby enhancing the validity of our findings.

These findings have significant clinical implications, suggesting that NA should be preferred over GA in urgent and emergency CS whenever feasible to minimize adverse neonatal outcomes, in line with current recommendations (1, 20). This recommendation is particularly relevant for situations where labor analgesia with an epidural catheter is already in place, as the use of the catheter allows rapid conversion to NA without the need for GA (21–23). Moreover, neonatologists must be prepared for proper resuscitation and increased NICU respiratory care, when GA is served in non-elective CS.

### 4.1 Limitations and future directions

The single-center and the retrospective design may limit the generalization of our findings. Additionally, the relatively small sample size for the GA group might affect the power to detect differences in some outcomes, particularly those with lower incidence rates. Future multicenter studies with larger sample sizes are needed to confirm our findings and further explore the long-term effects of GA on neonatal health. Due to the extreme imbalance in the use of GA in elective cesarean section cases, these cases were excluded from our analyses. The highly skewed distribution of the treatment variable posed a significant risk of introducing bias, making it challenging to achieve proper group balance, even with IPTW. Despite this limitation, future studies with larger cohorts—where advanced statistical methods could better account for group imbalances—are highly recommended to achieve a more comprehensive understanding of the impact of general anesthesia on neonatal outcomes. Moreover, time-sensitive analyses are needed to better elucidate the temporal aspects of this relationship and its correlation with the pharmacokinetics of anesthetic agents. The potential influence of additional confounding factors—such as the time interval from anesthesia administration to delivery, which was not currently available in our dataset—may further contribute to the detrimental effects of GA on neonatal outcomes. Future research should prioritize prospective multicenter studies with a focus on time-sensitive data collection to validate our findings and explore the underlying mechanisms driving the adverse effects of GA on neonates. The absence of neurological outcomes and other potential anesthesia-related complications in our cohort represents a key limitation of this study. Additionally, the lack of long-term follow-up data on developmental and neurocognitive outcomes further limits our findings. Future research efforts aimed at collecting such data are essential to gain a more comprehensive understanding of the potential long-term effects of general vs. neuraxial anesthesia on neonatal development and to fully assess the implications of anesthesia choice in CS cases.

## 5 Conclusion

In conclusion, our study provides robust evidence that general anesthesia is associated with significantly worse neonatal outcomes compared to neuraxial anesthesia in urgent and emergency cesarean sections. The novelty of our work lies in demonstrating that the impact of general anesthesia extends beyond delivery room challenges to the NICU phase, contributing to sustained neonatal morbidity. Furthermore, by detailing the specific resuscitation maneuvers required in neonates delivered under general anesthesia, our study provides actionable insights that enhance clinicians preparedness and ability to manage these high-risk situations effectively. These findings emphasize the critical importance of anesthesia choice in high-risk deliveries, advocating for the preference of neuraxial techniques to improve neonatal health outcomes and reduce the burden of intensive NICU interventions. Continued research is warranted to explore the long-term developmental consequences of general anesthesia exposure in these vulnerable populations.

## Data Availability

Anonymized data will be available to qualified academic investigators to replicate study results and as long as data transfer is in agreement with EU legislation on the general data protection regulation. Data transfer will be regulated by material transfer agreements and should be authorized by institutional Review Boards. Further enquiries can be directed to the corresponding author.
